# Pilot study of peripheral blood chemokines as biomarkers for atrial fibrillation-related thromboembolism and bleeding in elderly patients

**DOI:** 10.3389/fpubh.2022.844087

**Published:** 2022-09-23

**Authors:** Meihui Tai, Haiyan Shi, Hao Wang, Xiao Ma, Meng Gao, Qing Chang, Fang Li, Qiang Zeng, Yang Shi, Yutao Guo

**Affiliations:** ^1^Chinese PLA Medical College, Pulmonary Vessel and Thrombotic Disease, Sixth Medical Center, Chinese PLA General Hospital, Beijing, China; ^2^Department of Gastroenterology, Second Medical Center, National Clinical Research Center for Geriatric Diseases, Chinese PLA General Hospital, Beijing, China; ^3^Department of Cardiology, Second Medical Center, National Clinical Research Center for Geriatric Diseases, Chinese PLA General Hospital, Beijing, China; ^4^Health Management Institute, Second Medical Center, National Clinical Research Center for Geriatric Diseases, Chinese PLA General Hospital, Beijing, China

**Keywords:** atrial fibrillation, chemokine, elderly, thromboembolism, bleeding

## Abstract

**Background:**

The scoring systems currently used to identify the potential for thrombosis and bleeding events in high-risk atrial fibrillation patients have certain limitations. The aim of this pilot study was to identify inflammatory chemokines with potential utility as sensitive biomarkers for the risk of thrombosis and bleeding in elderly patients with non-valvular atrial fibrillation.

**Methods:**

From January 1, 2014, to December 31, 2017, 200 consecutive elderly patients with atrial fibrillation (average age: 87.6 ± 7.7 years) were enrolled and followed up for 2 years to observe thromboembolic (arterial and venous) and bleeding events. Serum was collected upon enrollment, and the baseline levels of 27 chemokines were analyzed. During the 2-year follow-up, 12 patients were lost to follow-up. Among the 188 patients, there were 32 cases (17.0%) of AF-related thrombosis, 36 cases (19.1%) of arterial thrombosis, and 35 cases (18.6%) of major bleeding events.

**Results:**

Among 188 patients, 30 patients without clinical events (control group), 23 with arterial thrombosis, 15 with atrial fibrillation-related venous thromboembolism, and 12 with major bleeding were selected and randomly matched to compare chemokine levels. The baseline levels of interleukin-6, interleukin-10, vascular cell adhesion molecule-1, chemokine C-C-motif ligand, B-lymphocyte chemoattractant 1, interleukin-4, E-selectin, fractalkine, C-X-C motif chemokine 12, and granulocyte chemotactic protein 2 were found to differ statistically among the four groups (*p* < 0.05). Compared with that in the control group, the level of interleukin-4 in patients with atrial fibrillation-related thrombosis, arterial thrombosis, or major bleeding increased by 53-fold (0.53 vs. 0.01 pg/ml), 17-fold (0.17 vs. 0.01 pg/ml), and 19-fold (0.19 vs. 0.01 pg/ml), respectively. Compared with that in the control group, the level of interleukin-6 in patients with arterial thrombosis increased by six-fold (39.78 vs. 4.98 pg/ml).

**Conclusions:**

Among elderly patients with atrial fibrillation at high risk of thromboembolism and bleeding, the baseline levels of interleukin-6, interleukin-4, and E-selectin were significantly increased in those that experienced thrombosis and bleeding events during the 2-year follow-up, indicating that these chemokines may serve as potential biomarkers for an increased risk of thrombosis and bleeding in this population.

**Clinical trial registration number:**

ChiCTR-OCH-13003479.

## Background

The incidence of atrial fibrillation (AF) increases significantly with age (1, 2). In the last decade, the burden of AF and AF-related stroke/thrombotic events in China increased significantly, but the rate of anticoagulation therapy prescribed for patients with AF in China was significantly lower than that in Europe and America, especially among elderly patients (2, 3). The risk of ischemic stroke, AF-related venous thrombosis, and acute myocardial infarction might coexist in elderly patients with AF (4, 5). The risk of thromboembolism in patients with AF can be effectively reduced by anticoagulation therapy. However, the dysfunction of organs such as the liver and kidney, presence of other diseases such as hypertension, diabetes, and atherosclerosis, and use of multiple medications that predispose elderly patients with AF to concomitant thromboembolism and bleeding (6, 7), which often leads to death or severe disability, profoundly affects the quality of life (8).

The current clinical risk scoring systems for thromboembolism are CHADS_2_ [congestive heart failure, hypertension, age of 75 years, diabetes, stroke (doubled)] and CHA_2_DS_2_-VASc [congestive heart failure, hypertension, age of 75 years (doubled), diabetes mellitus, stroke or transient ischemic attack (doubled), vascular disease, age of 65–74 years, female sex)]; whereas that for bleeding is HAS-BLED [hypertension, abnormal renal/liver function, stroke, bleeding history or predisposition, labile international normalized ratio, elderly (>65 years), drugs/alcohol concomitance]. In these systems, there are overlaps among clinical factors such as hypertension, previous stroke, and age, and the utility of these systems for identifying thrombosis and bleeding risk in high-risk patients with AF is limited (9–11).

Previous studies showed that chemokines play an important role in inflammation and atherosclerosis (12, 13). However, the relationship between chemokines and the prognosis of AF remains unclear. It has been suggested that when AF is associated with a hypercoagulable state, the levels of some cytokines are elevated. Gizatulina et al. found that blood concentration of growth differentiation factor 15 (GDF-15) as a predictor of left atrial/left atrial appendage (LA/LAA) thrombosis in patients with non-valvular atrial fibrillation (14). A systematic review and meta-analysis including five studies involving 22,928 patients and concerning the association between IL-6 and thromboembolic events in AF showed that higher level of IL-6 in AF patients is related to long-term thromboembolic events including stroke (RR 1.44, CI 95% 1.09–1.90, *p* = 0.01) and a higher risk of long-term bleeding risk (RR 1.36, CI 95% 1.06–1.74, *p* = 0.02) (15). So Inflammatory chemokines might serve as sensitive biomarkers for guiding the clinical management of anticoagulation treatment (16–19). This prospective study aimed to identify chemokines with potential utility as sensitive biomarkers in patients with AF at high risk of thromboembolism and bleeding.

## Methods

### Study population

This was a prospective observational cohort study (clinical trial registration number: ChiCTR-OCH-13003479). Between January 1, 2014, and December 31, 2015, 200 consecutive elderly patients (all aged over 75 years) with non-valvular AF at the General Hospital of the Chinese People's Liberation Army were enrolled (average age: 87.6 ± 7.7 years). The study was approved by the Medical Ethics Committee of the General Hospital of the People's Liberation Army (approval number: S2013-064-02).

The inclusion criteria for patients in the AF group (including new onset, paroxysmal, persistent, and permanent AF, as confirmed by electrocardiogram or 24 h Holter monitor) were age ≥75 years and at least one AF attack recorded in the year prior to the study. Patients or patients' relatives provided written consent. The exclusion criteria were age ≤75 years, rheumatic heart disease, biological or mechanical valve replacement or mitral valve repair, serious uncontrolled infection, and an AF attack recorded more than 1 year before the study.

### Clinical data collection

Clinical data were collected at the baseline and during follow-up by experienced attending cardiologists. The baseline clinical data of enrolled patients were as follows: name, gender, age, type of AF (new onset, paroxysmal, persistent, or permanent), systolic blood pressure, diastolic blood pressure, smoking and drinking history, disease history (such as ischemic stroke, hypertension, diabetes, congestive heart failure, peripheral vascular disease, and renal/liver dysfunction), and medication status [including the use of antiplatelet drugs (aspirin and clopidogrel) and anticoagulation drugs (warfarin and novel oral anti-coagulants)]. Follow-up data including medication status and clinical endpoints were collected through medical records, telephone interviews, and questionnaires.

Two hundred elderly patients with AF were followed up for 2 years to observe thromboembolic (both arterial and venous thrombosis) and bleeding events. AF-related thrombotic events included ischemic stroke, lower extremity deep venous thrombosis, and thrombosis of other systems (including mesenteric thrombosis and venous catheter-related thrombosis). Arterial thrombosis was defined as acute myocardial infarction and unstable angina. Clinically relevant major bleeding events included a decrease in hemoglobin of more than 2 g/L, the need for a blood transfusion or hospitalization, bleeding in major organs, and fatal bleeding. Non-clinically relevant major bleeding events included chronic bleeding and a decrease in hemoglobin of <2 g/L. Minor bleeding events included subcutaneous hematoma, gastrointestinal bleeding (below the standard for major bleeding), and blood sputum (20). Data collection was completed for 188 patients; 12 patients were lost to follow-up, the loss to follow-up rate is 6%.

### Measurement of chemokine levels

Two milliliters of fasting blood was drawn from all patients in the early morning on the day of enrollment. Blood samples were collected using EDTA anticoagulant vessels and then centrifuged at 4,000 rpm for 8 min (room temperature). Plasma was separated and stored at −80°C for batch analysis. The Aimplex method (Beijing Kuang Bo Biotechnology Limited by Share Ltd., Beijing, China) was used to detect 27 chemokines of the chemokine family, including neutrophil activating protein-2 (NAP2), P-selectin, E-selectin, intercellular cell adhesion molecule-1 (ICAM-1), vascular cell adhesion molecule-1 (VCAM-1), platelet factor 4 (PF4), monokine induced by interferon-r (MIG), B-lymphocyte chemoattractant 1 (BCA-1), regulated on activation normal T cell expressed and secreted (RANTES), monocyte chemoattractant protein (MIP)-3beta, MIP-1beta, tumor necrosis factor (TNF)-alpha, interleukin (IL)-1beta, IL-2, IL-4, IL-6, granulocyte chemotactic protein 2 (GCP2), IL-10, epithelial neutrophil activating peptide 78 (ENA-78), interferon-inducible protein-10 (IP-10), monocyte chemoattractant protein-1 (MCP-1), thymus and activation-regulated chemokine (TARC), chemokine C-C-motif ligand (CCL19), stromal cell-derived factor 1 [(SDF1; also known as C-X-C motif chemokine 12 (CXCL12)], fractalkine, and interferon-inducible T cell alpha chemoattractant (I-TAC). For each inflammatory chemokine, the intragroup error was <6%, while the intergroup error was <8%.

### Statistics

Continuous variables were expressed as the median [interquartile range (IQR)] if the data were non-normally distributed, or as the mean ± standard deviation (SD; ± SD) if the data were normally distributed (Kolmogorov-Smirnov criteria). The Kruskal-Wallis test was used to compare non-parametric data. Two-way ANOVA was used to compare data with a normal distribution. Qualitative data were expressed as percentages. A *p* < 0.05 was considered statistically significant. Statistical analysis was performed using IBM SPSS Statistics version 19.0 (SPSS, Inc., Chicago, IL, USA).

## Results

### Baseline clinical characteristics and risk of thrombosis and bleeding

The clinical characteristics of 188 patients with AF are presented in Table 1. This cohort was predominantly elderly (aged 75–98 years, average age: 87.6 ± 7.7 years) male patients (92.6%). This cohort of elderly patients with AF was at high risk of thromboembolism [CHA_2_DS_2_-VASc (mean ± SD): 5.1 ± 1.6] and bleeding [HAS-BLED (mean ± SD): 3.4 + 1.2].

**Table 1:** Baseline clinical characteristics of 188 elderly patients with AF at high risk of thromboembolism and bleeding.

**Characteristic**	**Patients with AF (*n* = 188)**
Age, mean (SD) (years)	87.6 (7.7)
Male, *n* (%)	174 (92.55)
Systolic blood pressure, mean (SD) (mmHg)	133.82 (13.65)
Diastolic blood pressure, mean (SD) (mmHg)	69.91 (10.62)
Smoking history, *n* (%)	90 (47.87)
Drinking history, *n* (%)	88 (46.81)
**Type of AF**, ***n*** **(%)**	
New onset AF, *n* (%)	7 (3.72)
Paroxysmal AF, *n* (%)	117 (62.23)
Persistent AF, *n* (%)	13 (6.91)
Permanent AF, *n* (%)	51 (27.13)
**Comorbidities**	
Hypertension, *n* (%)	135 (71.81)
Coronary heart disease, *n* (%)	143 (76.06)
Old myocardial infarction, *n* (%)	38 (20.21)
Hyperlipidemia, *n* (%)	41 (21.81)
Type 2 diabetes, *n* (%)	64 (34.04)
Congestive heart failure, *n* (%)	44 (23.40)
Stroke, *n* (%)	84 (44.68)
Peripheral vascular disease, *n* (%)	26 (13.83)
Renal dysfunction, *n* (%)	48 (25.53)
Liver dysfunction, *n* (%)	4 (2.13)
Anemia, *n* (%)	41 (21.81)
Past bleeding history, *n* (%)	39 (65.42)
Current bleeding, *n* (%)	15 (7.99)
Risk of fall, *n* (%)	68 (36.17)
CHA_2_DS_2_-VASc, mean (SD)	5.1 (1.6)
HAS-BLED, mean (SD)	3.4 (1.2)
**Anticoagulation therapy**	
Warfarin, *n* (%)	10 (5.32)
Rivaroxaban, *n* (%)	25 (13.30)
Clopidegrel, *n* (%)	78 (41.49)
Aspirin, *n* (%)	74 (39.36)

AF, atrial fibrillation; SD, standard deviation; CHA2DS2-VASc: congestive heart failure, hypertension, age 75 years (doubled), diabetes mellitus, stroke or transient ischemic attack (doubled), vascular disease, age 65 to 74 years, and female sex; HAS-BLED: hypertension, abnormal renal/liver function, stroke, bleeding history or predisposition, labile international normalized ratio, elderly (65 years), drugs/alcohol concomitance.

Among 188 elderly patients with AF, the number of patients on warfarin, rivaroxaban, clopidegrel, and aspirin were 10 (5.32%), 25 (13.30%), 78 (41.49%), and 74 (39.36%) respectively.

### Clinical outcomes of elderly patients with AF during follow-up

During the 2-year follow-up, among the 188 patients, there were 32 cases (17.0%) of AF-related thrombosis (including ischemic stroke, deep-vein thrombosis, and thrombosis of other systems), 36 cases (19.1%) of arterial thrombosis,35 cases (18.6%) of major bleeding events (including hemorrhagic stroke and other clinical major bleeding events), and 36 cases (19.2%) of minor bleeding. There were 54 cases (28.7%) of all-cause death (Table 2).

**Table 2:** Adverse clinical events in 188 elderly patients with AF at high risk of thromboembolism and bleeding during 2-year follow-up.

**Adverse event**	**Case #**	**Incidence (%)**
**AF-related thromboembolism**		
Ischemic stroke	8	4.26
Lower extremity deep venous thrombosis	19	10.11
Other-system thrombosis	5	2.66
Arterial thrombosis		
Acute myocardial infarction	22	11.70
Unstable Angina	14	7.45
**Bleeding**		
Cerebral hemorrhage	8	4.26
Clinically relevant major bleeding	18	9.57
Non-clinically relevant major bleeding	9	4.79
Minor bleeding	36	19.15
All-cause death	54	28.72

AF, atrial fibrillation; Other-system thrombosis: mesenteric thrombosis, venous catheter-related thrombosis, etc.; Clinically relevant major bleeding: hemoglobin decreased more than 2 g/L, the need for blood transfusion or hospitalization, or bleeding in major organs, fatal bleeding, etc.; Non-clinically relevant major bleeding: chronic bleeding, hemoglobin decreased less than 2 g/L; Minor bleeding: subcutaneous hematoma, gastrointestinal bleeding (less than the standards of major bleeding), blood sputum, etc.

### Association between chemokines and AF-related arterial thrombosis, venous thrombosis, and bleeding events

Thirty high-risk patients with AF but not exhibiting clinical adverse events (control group), 23 with arterial thrombosis (unstable angina and acute myocardial infarction), 15 with AF-related venous thrombosis, and 12 with major bleeding were selected and matched randomly to analyze the 27 chemokines. The baseline levels of IL-6, IL-10, VCAM-1, CCL19, BCA-1, IL-4, E-selectin, fractalkine, CXCL12, and GCP2 were found to differ statistically among the control, arterial thrombosis, AF-related venous thrombosis, and major bleeding groups (*p* < 0.05). Compared with those in the control group, the levels of IL-4 in patients with AF-related venous thrombosis, arterial thrombosis, or major bleeding events increased by 53- (0.53 vs. 0.01 pg/ml), 17- (0.17 vs. 0.01 pg/ml) and 19-fold (0.19 vs. 0.01 pg/ml), respectively. Compared with those in the control group, the levels of IL-6 in patients with arterial thrombosis increased by six-fold (39.78 vs. 4.98 pg/ml; Table 3).

**Table 3:** Levels of chemokines in elderly patients with AF and different adverse clinical events.

**Chemokine**	**Control**		**Arterial thrombosis**		**AF related venous thromboembolism**		**Major bleeding**		***p***
	**(*****n*** = **30)**		**(*****n*** = **23)**		**(*****n*** = **15)**		**(*****n*** = **12)**		
IL-6, pg/ml	4.98	(3.05–10.49)	9.21	(3.75–20.82)	13.86	(7.36–21.81)	39.78	(10.06–182.96)	**<0.001**
IL-10, pg/ml	3.73	(3.24–4.78)	4.52	(3.98–6.64)	4.75	(3.28–7.35)	6.31	(4.01–15.00)	**0.01**
VCAM-1, ng/ml	358.53	(292.39–474.19)	438.93	(282.19–463.42)	468.53	(325.93–561.71)	643.97	(374.09–1079.54)	**0.02**
CCL19, pg/ml	95.25	(66.70–109.48)	106.21	(76.52–133.43)	99.41	(72.17–143.85)	132.18	(97.36–196.46)	**0.03**
BCA-1, pg/ml	5.54	(3.96–9.18)	6.31	(4.34–10.11)	6.55	(5.06–13.86)	9.41	(54.61–5.68)	**0.04**
IL-4, pg/ml	0.01	(0.01–0.17)	0.19	(0.01–1.90)	0.53	(0.02–1.67)	0.17	(0.01–0.93)	**0.04**
E-selectin, ng/ml	8.59	(5.62–13.06)	9.94	(5.92–12.76)	5.34	(3.74–11.79)	15.52	(8.37–21.94)	**0.04**
Fractalkine, ng/ml	32.16	(24.51–37.63)	34.14	(27.28–45.05)	35.24	(24.96–44.69)	51.12	(32.18–101.47)	**0.05**
CXCL12, pg/ml	2.32	(1.57–4.48)	2.84	(1.85–6.52)	3.91	(2.80–10.49)	4.16	(3.04–9.23)	**0.05**
GCP2, pg/ml	22.22	(16.42–33.03)	23.93	(20.24–28.40)	25.81	(23.71–28.95)	32.07	(25.39–45.66)	**0.05**
MCP-1, pg/ml	73.27	(51.28–93.71)	74.43	(56.64–92.80)	71.53	(64.98–98.77)	124.82	(80.63–193.93)	0.06
PF4, ng/ml	6.62	(0.44)	6.49	(0.68)	6.65	(0.42)	7.04	(1.23)	0.08
IP-10, pg/ml	36.34	(25.66–50.51)	33.16	(24.17–49.65)	40.79	(36.42–73.35)	48.82	(38.67–59.12)	0.11
TNF-alpha, pg/ml	0.13	(0.01–0.57)	0.37	(0.01–1.03)	0.01	(0.01–1.57)	0.31	(0.01–1.12)	0.12
I-TAC, pg/ml	6.16	(4.26–15.60)	6.01	(4.57–9.47)	6.92	(4.10–16.77)	6.59	(3.72–15.61)	0.12
MIG, ng/ml	0.28	(0.08)	0.27	(0.07)	0.28	(0.10)	0.34	(0.11)	0.14
ICAM-1, ng/ml	282.25	(113.22)	310.42	(156.41)	326.69	(134.32)	379.35	(160.31)	0.17
IL-2, pg/ml	3.26	(2.48–3.69)	3.08	(2.51–4.49)	3.44	(2.85–4.49)	3.21	(2.16–5.40)	0.23
IL-1beta, pg/ml	0.02	(0.01–0.02)	0.02	(0.02–0.02)	0.02	(0.02–0.02)	0.02	(0.02–2.51)	0.27
IL-8, pg/ml	61.48	(23.36–113.33)	59.12	(27.01–156.94)	97.03	(48.58–213.75)	112.39	(66.39–199.48)	0.43
NAP2, ng/ml	18.26	(3.29)	17.79	(3.94)	16.54	(4.68)	18.01	(3.67)	0.54
ENA-78, ng/ml	1.55	(1.03)	1.59	(1.08)	1.66	(0.83)	1.24	(0.86)	0.59
MIP-1alpha, pg/ml	20.59	(17.44–37.44)	29.17	(13.84–38.16)	26.95	(25.58–45.28)	41.70	(28.20–53.25)	0.67
MIP-3beta, pg/ml	92.69	(27.91)	106.95	(44.31)	114.26	(47.65)	175.61	(137.92)	0.75
P-selectin, ng/ml	80.95	(28.23)	82.81	(29.37)	77.47	(32.47)	87.00	(34.90)	0.79
TARC, pg/ml	41.52	(18.99–74.24)	44.42	(18.54–93.56)	31.28	(9.99–108.72)	30.01	(14.52–83.02)	0.82
RANTES, ng/ml	2.34	(0.29)	2.24	(0.33)	2.38	(0.45)	2.75	(1.02)	0.84

AF, atrial fibrillation; IL, interleukin; VCAM-1, vascular cell adhesion molecule-1; CCL19, chemokine C–C-motif ligand; BCA-1, B-lymphocyte chemoattractant 1; CXCL12, C–X–C motif chemokine 12, also known as stromal cell-derived factor 1 (SDF1); GCP2, granulocyte chemotactic protein 2; MCP-1, monocyte chemoattractant protein-1; PF4, platelet factor 4; IP-10, interferon–inducible protein-10; TNF–alpha, tumor necrosis factor-alpha; I-TAC, interferon-inducible T cell alpha chemoattractant; MIG, monokine induced by interferon-r; ICAM-1; intercellular cell adhesion molecule-1; NAP2, neutrophil activating protein-2; ENA-78, epithelial neutrophil activating peptide 78; MIP, monocyte chemoattractant protein; TARC, thymus and activation–regulated chemokine; RANTES, regulated on activation normal T cell expressed and secreted. Median ± quartile: IL-6, IL-10, VCAM-1, CCL19, BCA-1, IL-4, E-selectin, fractalkine, CXCL12, GCP2, MCP-1, IP-10, TNF-alpha, I-TAC, IL-2, IL-1beta, IL-8, MIP-3beta, TARC. Mean ± standard deviation: PF4, MIG, ICAM-1, NAP2, ENA-78, MIP-1alpha, P-selectin, RANTES. Continuous variables were expressed as the median [interquartile range (IQR)] if the data were non-normally distributed, or as the mean ± standard deviation (SD; — X± SD) if the data were normally distributed (Kolmogorov-Smirnov criteria). The bold value represents *p* < 0.05.

## Discussions

### Chemokines and AF-related stroke

Accumulating data indicate that endothelial dysfunction, platelet activation, and coagulation factor activation caused by inflammation might cause patients with AF to enter a prothrombotic state (21–23). The upregulation of inflammatory factors such as IL-6 and IL-8 are related to endothelial dysfunction, platelet activation, and active thrombosis (21, 22). Experimental studies have demonstrated that the IL-6-induced expression of tissue factor and von Willebrand factor is involved in platelet activation, possibly driven by platelet-monocyte interactions (24, 25). Patients with increased levels of IL-6 are more likely to have thrombus formation in the left atrial appendage (21, 22). Moreover, Pinto et al. (26) found that elevated levels of IL-6, von Willebrand factor, and TNF-alpha are significant predictors of stroke, based on a 3-year follow-up of 373 patients with AF. IL-6 is also an independent predictor of the composite endpoint of stroke or death in patients with AF (27) and is associated with worse outcomes after ischemic stroke (28).

### IL-6 and AF-related thrombosis

The patients with AF in our study were at high risk of both thromboembolism and bleeding and were much older than the patients enrolled in these previous studies. We found that the levels of IL-6 increased significantly in patients with arterial thrombosis and AF-related thrombosis during the 2-year follow-up; our results were consistent with the previous findings. Interestingly, we also found that the expression of IL-6 was significantly increased in patients with AF with major bleeding. This result was consistent with the findings of the RE-LY (Randomized Evaluation of Long-Term Anticoagulation Therapy Study) trial, in which an independent relationship was identified between IL-6 and a higher risk of stroke and major bleeding independent of clinical risk factors, even after adjusting for cardiovascular biomarkers to attenuate the prognostic value (29).

### E-selectin/VCAM-1 and AF-related thrombosis/bleeding

Under normal physiological conditions, soluble E-selectin (sE-sel) is not expressed on the surface of endothelial cells (30). Under pathological conditions, increased expression of E-selectin on the surface of endothelial cells can mediate the activation of endothelial cells and the adhesion of leukocytes, which may play a role in the process of thrombosis (31). However, data regarding whether sE-sel contributes to the risk of thromboembolism in patients with AF remains limited and confusing. For instance, Freestone et al. (32) found that compared to patients with sinus rhythm, patients with AF have elevated levels of sE-sel, and low baseline sE-sel levels predict the successful maintenance of sinus rhythm at 6 months of follow-up (33). In contrast, another study conducted by Freestone et al. (34) indicated that in patients with systolic heart failure, the differences between plasma sE-sel levels in patients with AF and those in patients with normal sinus rhythm were not significant.

Similar to the first study by Freestone et al., a study by Krishnamoorthy et al. (35) of 423 patients with non-valvular AF followed up for 19 months showed that elevated levels of sE-sel were associated with an increased risk of clinical adverse events (acute myocardial infarction, ischemic stroke, and all-cause mortality). However, Roldán et al. (36) found no association between sE-sel levels and inflammation or abnormal thrombogenesis in 191 consecutive patients with chronic non-rheumatic AF who were not receiving anticoagulation therapy. However, plasma levels of sE-sel were significantly decreased (*p* < 0.01) after oral anticoagulation therapy.

In the Bruneck study (37), a prospective population-based cohort study with a 20-year follow-up (*n* = 909), the levels of soluble VCAM-1 were significantly associated with incident AF (hazard ratio: 1.49; 95% confidence interval, CI: 1.26–1.78, *p* < 0.001 with a Bonferroni correction for both values); even after adjusting for age and sex, the association was still significant. However, the association with E-selectin levels was insignificant (after a Bonferroni correction in unadjusted and age- and sex-adjusted analyses).

Our results showed that the levels of E-selectin were significantly increased in the major bleeding and AF-related thrombosis groups, compared with those in patients without thromboembolic and bleeding events. Furthermore, the levels of VCAM-1 were different significantly among control group vs. arterial thrombosis, venous thrombosis, and major bleeding groups (*p* < 0.05). Further *in vivo* and *in vitro* studies are needed to investigate whether increased E-selectin levels, which reflect endothelial activation and platelet aggregation, initiate AF and AF-related thrombosis or are simply a consequence of AF-accompanied cardiovascular disease and adverse clinical events.

### IL-4/IL-10 and AF-related thrombosis/bleeding

IL-4 and IL-10, secreted by type II T helper lymphocytes (Th2 lymphocytes), can inhibit the synthesis and expression of proinflammatory markers such as IL-6, IL-8, and TNF by inhibiting type I T helper lymphocytes and stimulating B and Th2 lymphocytes, thus reducing the inflammatory response (38). The IL-10-592A/C polymorphism is associated with AF, and people of Han Chinese descent with the A allele are at an increased risk of AF (39). Henningsen et al. (30) did not find significant differences in either the genotypic or allelic frequency of IL-10-1082 between patients with AF and the control group, although the expression of inflammation and cell adhesion molecules in a rat model of venous thrombosis was inhibited by viral IL-10 gene transfer (40), and the IL-10-1082A/G polymorphism is associated with the risk of deep venous thrombosis (41). A C582T polymorphism in the IL-4 gene [odds ratio, OR ¼: 1.40, 95% CI: 1.13–1.73, *p* ¼: 0.003)] was found to be an independent predictor of thromboembolic stroke (42).

In our study, we did observe significantly higher baseline levels of IL-4 and IL-10 in patients with thromboembolic or bleeding events during the 2-year follow-up, compared to those in patients without these adverse events. Especially, compared to those in the patients without adverse events, the baseline levels of IL-4 in patients with venous thrombosis during follow-up increased by 53-fold. It suggested that IL-4 and IL-10 may highly be associated with the progression of AF-related adverse events. However, IL-4 levels could be associated with some medical setting, such as scleroderma, multiple sclerosis, chronic sinusitis, inflammatory bowel diseases, bronchial asthma, atopic dermatitis (43–45). Although we excluded patients with rheumatic heart disease and serious uncontrolled infection, other disease didn't be excluded, which needed to be explored further.

### Possible mechanisms

There is a close relationship between inflammation, platelet activation and AF. Inflammatory cytokines play a certain role in the maintenance and prognosis of AF (46). Inflammation leads to cardiac endothelial damage or dysfunction, and promotes the aggregation of platelets, and the activation fibrinolytic protein and clotting factors (47). At the same time, the reconstruction of atrial anatomy cause by AF provides the environment for platelet aggregation and blood coagulation which promotes thrombosis (48). Chemokines, to a certain extent, is involved in inflammation, angiogenesis and necrosis, which is a key mediator of inflammation-related cell migration (49). Studies suggests that pro-inflammatory cytokines, such as IL-1β, IL-6, and IL-8, promote a pro-coagulant state through the induction of tissue factor. Anti-inflammatory cytokines, including IL-2, IL-4, and IL-10, act to reduce coagulation induced by other pro-inflammatory factors (50). Additionally, the cytokines IL-4, IL-6, IL-8, IL-10, and IL-1β were all significantly elevated in massive PE patients in comparison to those with low-risk and submassive PE, indicating the overwhelming inflammatory response with larger embolism (51). It is found that the polymorphisms of IL-4−589C/T and IL-13 intron 3C/T were associated with the increased risk of VTE among women, while IL-6 174C/G was found to make men susceptible to the VTE event (51). When compared with patients with lacunar infarct, those with cardioembolic stroke were shown to have significantly increased levels of proinflammatory cytokines such as IL-1β and IL-6 (51). The mechanisms of chemokines with increased levels in patients that experienced thrombosis and bleeding events during the 2-year follow-up in our study should be further studied *in vivo* and *in vitro*.

## Limitations

There were several limitations in this study. Although there were 18.6% patients on anticoagulation, the anticoagulant may impact the thromboembolism and bleeding event. Moreover, this was single-center study and most of patients enrolled in Beijing, the result cannot be generalized other population. Given the small sample in this study, there were not exactly matched for age and sex, which possibility caused the bias. In addition, we were unable to perform multivariate regression analysis after adjusting the confounders. The temporal changes in the chemokines and their effect on clinical outcomes in patients with AF will be examined in our future analysis. Additionally, we did not compare differences in chemokines among different types of AF, due to the limited number of subjects.

## Conclusion

In summary, among the 27 chemokines screened in our study, IL-6, IL-4, and E-selectin exhibited significantly increased levels in the study group that experienced thrombosis and bleeding events.

## Data availability statement

The raw data supporting the conclusions of this article will be made available by the authors, without undue reservation.

## Ethics statement

This study was approved by Medical Ethics Committees of the Chinese PLA General Hospital (Reference Number: S2013-064-02). The patients/participants provided their written informed consent to participate in this study.

## Author contributions

YG conceived and designed the study. YG, HW, and MT analyzed and interpreted the data and wrote the report. YG supervised the work. MT and HW engaged in cohort assignment for participants. MT, HS, HW, XM, MG, QC, FL, QZ, YS, and YG reviewed and interpreted the results, commented on the report, contributed to revisions, and read and approved the final version. All authors contributed to the article and approved the submitted version.

## Funding

This work was supported by the National Natural Science Foundation of China (Reference Number: 81872920).

## Conflict of interest

The authors declare that the research was conducted in the absence of any commercial or financial relationships that could be construed as a potential conflict of interest.

## Publisher's note

All claims expressed in this article are solely those of the authors and do not necessarily represent those of their affiliated organizations, or those of the publisher, the editors and the reviewers. Any product that may be evaluated in this article, or claim that may be made by its manufacturer, is not guaranteed or endorsed by the publisher.

## Abbreviations

AF: atrial fibrillation
CHADS2: congestive heart failure, hypertension, age of 75 years, diabetes, stroke (doubled)
CHA2DS2-VASc: congestive heart failure, hypertension, age of 75 years (doubled), diabetes mellitus, stroke or transient ischemic attack (doubled), vascular disease, age of 65 to 74 years, female sex
HAS-BLED: hypertension, abnormal renal/liver function, stroke, bleeding history or predisposition, labile international normalized ratio, elderly (> 65 years), drugs/alcohol concomitance
NAP2: neutrophil activating protein-2
ICAM-1: intercellular cell adhesion molecule-1
VCAM-1: vascular cell adhesion molecule-1
PF: platelet factor
MIG: monokine induced by interferon-r
BCA-1: B-lymphocyte chemoattractant 1
RANTES: regulated on activation normal T cell expressed and secreted
MIP: monocyte chemoattractant protein
TNF: tumor necrosis factor
IL: interleukin
GCP2: granulocyte chemotactic protein 2
ENA-78: epithelial neutrophil activating peptide-78
IP-10: interferon-inducible protein-10
MCP-1: monocyte chemoattractant protein-1
TARC: thymus and activation-regulated chemokine
CCL19: chemokine C-C-motif ligand
SDF1: stromal cell-derived factor 1
CXCL12: C-X-C motif chemokine 12
I-TAC: interferon-inducible T cell alpha chemoattractant
sE: selsoluble E-selectin
Th2 lymphocytes: T helper lymphocytes.
